# Photodegradation Pathways of Typical Phthalic Acid Esters Under UV, UV/TiO_2_, and UV-Vis/Bi_2_WO_6_ Systems

**DOI:** 10.3389/fchem.2019.00852

**Published:** 2019-12-13

**Authors:** Chunying Wang, Ting Zeng, Chuantao Gu, Sipin Zhu, Qingqing Zhang, Xianping Luo

**Affiliations:** ^1^College of Resource and Environmental Engineering, Jiangxi University of Science and Technology, Ganzhou, China; ^2^School of Chemistry and Chemical Engineering, Xiamen University, Xiamen, China

**Keywords:** phthalic acid esters, UV, UV/TiO_2_, UV-Vis/Bi_2_WO_6_, degradation pathways

## Abstract

Photolysis and photocatalysis of typical phthalic acid esters (dimethyl phthalate, DMP; diethyl phthalate, DEP; dibutyl phthalate, DBP) were carried out in UV, UV/TiO_2_, and UV-Vis/Bi_2_WO_6_ systems. All of the selected phthalic acid esters and their decomposition byproducts were subjected to qualitative and quantitative analysis through HPLC and GC-MS. The results of 300 min of photolysis and photodegradation reaction were that each system demonstrated different abilities to remove DMP, DEP, and DBP. The UV/TiO_2_ system showed the strongest degradation ability on selected PAEs, with removal efficiencies of up to 93.03, 92.64, and 92.50% for DMP, DEP, and DBP in 90 min, respectively. UV-Vis/Bi_2_WO_6_ had almost no ability to remove DMP and DEP. However, all of the systems had strong ability to degrade DBP. On the other hand, the different systems resulted in various byproducts and PAE degradation pathways. The UV system mainly attacked the carbon branch and produced o-hydroxybenzoates. No ring-opening byproducts were detected in the UV system. In the photocatalytic process, the hydroxyl radicals produced not only attacked the carbon branch but also the benzene ring. Therefore, hydroxylated compounds and ring-opening byproducts were detected by GC-MS in both the UV/TiO_2_ and UV-Vis/Bi_2_WO_6_ photocatalytic systems. However, there were fewer products due to direct hole oxidation in the UV-Vis/Bi_2_WO_6_ system compared with the UV/TiO_2_ system, which mainly reacted with the pollutants via hydroxyl radicals.

## Introduction

Phthalic acid esters (PAEs), or phthalates, a chemical class of plasticizers, are widely used in plastic products. The consumption of PAE-containing products results in PAE release to water, the atmosphere, and the soil environment (Rastkari et al., 2018), and PAEs were detected everywhere. After an exploration of sources, Jiang suggested that the discharge of domestic wastewater may be a primary contributor to the occurrence of phthalate monoesters in Taihu Lake (Jiang et al., 2018). There was a maximum detection of 2,497 μg/L dibutyl phthalate (DBP) in the influent of a wastewater treatment plant in Africa. Different treatment processes had different removal effects, and DBP was shown to tend to accumulate in the sludge, exceeding acceptable levels for a safe aquatic environment (Salaudeen et al., 2018). Noura (Al-Jandal et al., 2018) revealed significant levels of phthalates in seawater (2.1–4.6 μg/L) and sediment (2.1–15.7 mg/kg dry wt) samples. They deduced that the phthalates were from wastewater treatment plants due to detections in outflow samples. Ozturk also reported that sewage effluents and sludge are identified as significant routes for the introduction of DEHP into the environment (Ozturk, 2018). Phthalates have even been detected in landfill leachate (Aarthi et al., 2018). It was revealed that diethyl phthalate (DEP), DBP, and DEHP were the major phthalates present in the groundwater of the karst region of northern Puerto Rico (Torres et al., 2018). After the detection of semi-volatile compounds in cloud waters, it was found that phthalates were the primary pollutants (Lebedev et al., 2018 source: cloud). Ecological security assessment of plastic greenhouse soil found it to be contaminated by phthalate esters (Zhou et al., 2018). DMP, DEP, and DBP were all detected in drinking water drawn from taps in Lagos, Nigeria (Dada and Ikeh, 2018). Meanwhile, researchers detected high levels of typical phthalates, including di-isobutyl phthalate (DiBP), DBP, Di(2-ethilhexyl) phthalate (DEHP), and DEP, in both Chinese and French hospitals (Wang et al., 2015; Baures et al., 2018).

PAEs are ubiquitous in various environments. As we know, PAEs are a typical type of endocrine-disrupting chemical and pose potential threats to the environment and humans. DBP, DEP, and DEHP have been reported to be the compounds that contribute most to different health effects after exposure to the residential indoor environment via ingestion, inhalation, and skin absorption, especially in children (Pelletier et al., 2018; Weiss et al., 2018). Li's research illustrated that the PAEs can also react with pollutants in outdoor air and contribute secondary organic aerosols (Li et al., 2018). DMP, DEP, DIBP, DNBP, and DEHP were the most detected PAEs from hair and dust in Chongqing, China, and the concentration of PAEs was higher in rural samples than that in urban samples due to the extensive usage of plastic film in rural areas (He et al., 2018). Xiao Kong's study indicated that DBP pollution could increase the health risk from vegetables and alter the biodiversity of indigenous bacteria in soil-vegetable ecosystems, which might further effect ecosystem functions in agricultural fields (Kong et al., 2018). For example, DEHP posed a severe threat to root tissues of wheat at the seedling and jointing stages (Gao et al., 2018). Many PAEs are demonstrated to be toxic to reproduction, impair development in aquatic animals (amphibians and crustaceans), and induce genetic mutation (Yin et al., 2018). DEHP can cause hepatotoxicity in quails via triggering nuclear xenobiotic receptors and modulating cytochrome P450 systems (Zhang Y.-Z. et al., 2018). DBP increased apoptosis, necrosis, and head malformation in *Xenopus* embryos (Xu and Gye, 2018). DiNP (diisononyl phthalate) disrupted the endocannabinoid system of zebrafish and affected reproduction in a gender-specific manner (Forner-Piquer et al., 2018). More sadly, exposure to PAEs also might result in recurrent pregnancy loss in reproductive-aged women (Liao et al., 2018).

DMP, DEP, and DBP are representatives of the short-chain phthalates. They are absorbed readily through the skin and the digestive tract and are then distributed to most tissues, even the placenta, as mentioned above. It has been reported that PAEs with alkyl chain lengths of <6 have intrinsic toxicity to aquatic organisms (Staples et al., 1997). All of the research and studies into the distribution and toxicity reminds us to pay more attention to the removal and degradation of these typical, widely used PAEs. Biodegradation (Ahuactzin-Perez et al., 2018), physical adsorption (Shaida et al., 2018), and chemical oxidation (Vela et al., 2018) have all been adopted to investigate the treatment of PAEs in the aquatic environment. Chemical oxidation is paid more attention due to the fast reaction and complete decomposition, especially the advanced oxidation process (AOPs). As one of the most promising AOPs, photocatalytic oxidation technology (Xu et al., 2015; Song et al., 2016) often possesses advantages in terms of mineralization or control of cytotoxicity compared with other AOPs. TiO_2_ is the most widely studied catalyst (Kumar and Devi, 2011), while Bi_2_WO_6_ has been researched more as a visible-light-driven material (Zhang K. et al., 2018). Our group has carried out a great deal of work to research the photocatalytic activities of TiO_2_ and Bi_2_WO_6_ (Luo et al., 2015, 2017; Wang et al., 2016; Wang C. et al., 2019). Herein, DMP, DEP, and DBP were chosen as typical PAEs to distinguish the degradation pathways of PAEs in UV, UV/catalyst, and UV-Vis/catalyst systems. TiO_2_ and Bi_2_WO_6_ were selected as the photocatalysts for UV/catalyst and UV-Vis/catalyst systems, respectively, due to the extensive research on and mature synthesis method of these two materials.

## Materials and Reagents

### Reagents

Standard solutions of DMP (1,000 mg/L in methanol), DEP (1,000 mg/L in methanol), and DBP (68.8 mg/L in methanol) were purchased from the Ministry of Environmental Protection Standard Samples Institute, China. Dichloromethane, acetonitrile, and anhydrous magnesium sulfate were bought from J&K Scientific LTD, Beijing. All of the purchased reagents were used without further purification. The photocatalysts TiO_2_ and Bi_2_WO_6_ were prepared by the sol-gel method and hydrothermal method, respectively, as reported in previous research (Wang et al., 2016; Wang C. et al., 2019).

### Photolysis and Photocatalysis Tests

The photolytic experiments were conducted using a 500 W mercury lamp to emit ultraviolet light (the main wavelength is 365 nm). Photocatalytic degradation was divided into the following two systems: UV/TiO_2_ system, 500 W mercury lamp (with maximum emission at 365 nm, and a light intensity of about 7.28 mW/cm^2^) as the light source and TiO_2_ as the photocatalyst; UV-Vis/Bi_2_WO_6_ system, 500 W xenon lamp (simulated solar irradiation, and a light intensity of about 4.2 mW/cm^2^) as the light source and Bi_2_WO_6_ as the photocatalyst. Both photolysis and photocatalytic degradation were carried out for 300 min in a photochemical reaction apparatus (XPA-7, Xujiang Electromechanical Plant, Nanjing, China). The temperature of the reaction systems was maintained at 20 ± 2°C by circulating water. The amount of catalyst was 1.0 g/L. Standard solutions of DMP, DEP, and DBP were used after dilution by deionized water without further pH adjustment, and the initial reaction concentrations of DMP, DEP, and DBP were 6, 6, and 4.128 mg/L, respectively.

### Analysis of PAEs and Their Intermediates

PAEs in the reaction solution were analyzed on an Agilent 1260 LC instrument equipped with a Quaternary HPLC pump, an Ultraviolet Detector (wavelength at 227), and a Zorbax Eclipse XDB-C18 column (4.6 × 150 mm, particle size 5 μm) kept at 25°C. The mobile phase was 60% acetonitrile and 40% pure water with a flow rate of 0.8 mL min^−1^.

The intermediates were extracted by methylene chloride from the aqueous solution. First of all, the reaction solution was extracted three times by 10 mL, 5 mL, and 5 mL of dichloromethane in sequence, and each extraction lasted for 10 min, with oscillation to ensure that the layer was obvious. After extraction, the extract was dried over anhydrous MgSO_4_, concentrated on a rotary evaporator, and then partitioned into 1 mL of liquid in vials with dichloromethane. Finally, 1 ul was injected into a GC/MS system (Agilent 5977A-7890B, USA) equipped with a HP-5MS Column. The initial temperature of the oven was 35°C. This was maintained for 2 min, then increased to 300°C at a rate of 8°C/min, and maintained for 10 min. The inlet temperature, GC-MS interface temperature, ion source temperature, and quadrupole temperature were 280, 250, 230, and 150°C, respectively. Helium was used as the carrier gas at 1.0 mL/min. The mass selective detector was operated in electron impact (EI) mode with an electron energy of 70 eV.

## Results and Discussion

### Removal of DMP, DEP, and DBP

**Figure 1:**
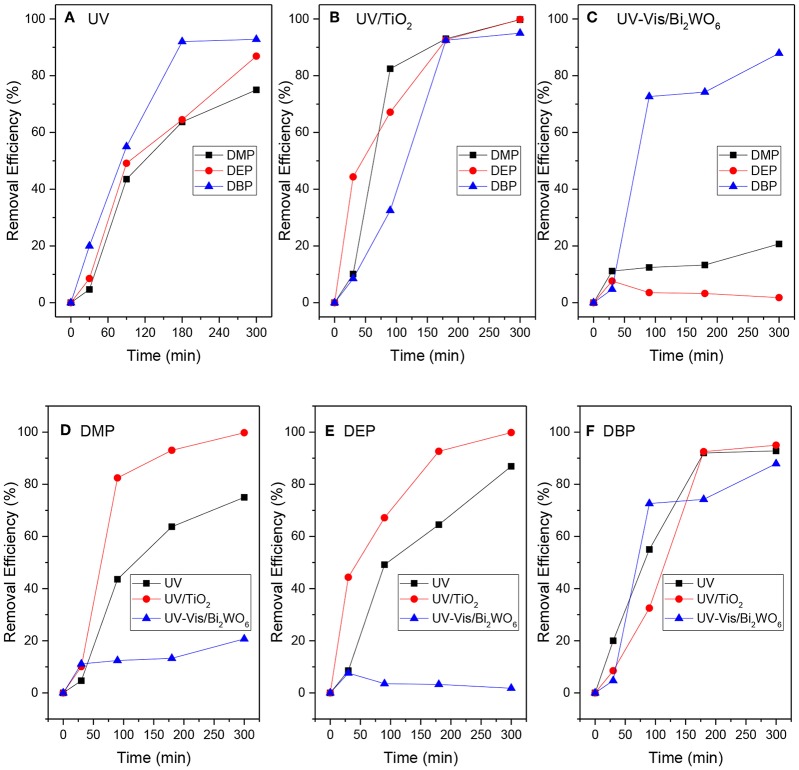
The removal of DMP, DEP, and DBP in different degradation systems (**A**: PAEs in the UV system; **B**: PAEs in the UV/TiO_2_ system; **C**: PAEs in the UV-Vis/Bi_2_WO_6_ system; **D**: DMP in the three systems; **E**: DEP in the three systems; **F**: DBP in the three systems).

DMP, DEP, and DBP are all homologues of phthalate, so they possess similar physical and chemical properties. However, the relative removal effects of the same degradation system or different degradation systems on the different phthalates were not known. The degradation characteristics of DMP, DEP, and DBP in UV, UV/TiO_2_, and UV-Vis/Bi_2_WO_6_ were investigated in detail, as illustrated in Figure 1.

The experiments indicated that TiO_2_ and Bi_2_WO_6_ had almost no adsorption ability to selected PAEs. As shown in Figure 1A, the degradation rate of PAEs increased with increase in the molecular weight or alkyl chain length in the UV system: DBP > DEP > DMP. This is consistent with the conclusion reached in the literature under 254 nm ultraviolet irradiation (Peng et al., 2013). However, the result in the UV/TiO_2_ system (Figure 1B) was completely different from that in the UV system. DEP and DMP were more liable to be degraded compared with DBP in the UV/TiO_2_ system. Furthermore, the DBP-removal efficiency was up to 90% after 300 min in the UV-Vis/Bi_2_WO_6_ system (Figure 1C), while there was almost no removal of DMP and DEP in that system. From another point of view, each system demonstrated different degradation abilities to DMP, DEP, and DBP. As indicated by Figures 1D,E, the UV/TiO_2_ system showed the strongest degradation ability on DMP and DEP, while UV-Vis/Bi_2_WO_6_ had almost no ability to degrade DMP and DEP. All of the systems had strong abilities to degrade DBP (Figure 1F). However, it is noteworthy that the DBP-degradation efficiency was higher than that of DEP in the persulfate system (Zhang D. et al., 2018; Wang Z. et al., 2019).

**Figure 2:**
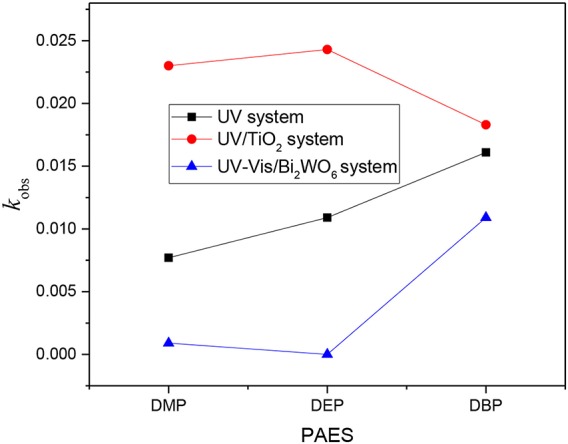
Reaction rate constants of PAEs in three degradation systems.

The following first-order reaction (Equation 1) (Badi et al., 2019) was employed to describe the degradation kinetics of PAEs in all of the experiments,

(1) $$\text{ln}({\text{C}}_{\text{t}}/{\text{C}}_{\text{0}})=k_{\text{obs}}\times t$$

where, *C*_t_ is the concentration of PAEs at any instant time *t, C*_0_ is the initial concentration of PAEs, and *K*_obs_ is the pseudo-first-order rate constant. Based on the linear relationship between ln (*C*_t_/*C*_0_) and time, the assumed first-order kinetics was conformed. The rate constants also reflect the above results, as shown in Figure 2.

### Analysis of Photodegradation Pathways of PAEs

#### DMP

**Figure 3:**
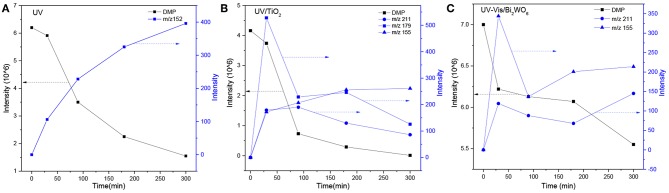
Time-dependent evolution profiles of DMP and its byproducts in different systems (**A**: UV; **B**: UV/TiO_2_; **C**: UV-Vis/Bi_2_WO_6_).

As the shortest-chain phthalate, the decomposition of DMP has been researched widely. Huang et al. (2013) demonstrated that UV/TiO_2_ had a stronger removal ability than photolysis at 365 nm, which is in agreement with this study, as discussed in section Removal of DMP, DEP, and DBP, but they only compared the removal efficiency without the byproducts. The byproducts of DMP and their time-dependent evolution profiles are shown in Table 1 and Figure 3.

**Table 1:** GC-MS results for DMP and its byproducts.

**Byproduct**	**m/z**	**Formula**	**Possible structure**	**Degradation system**
DMP	194	C_10_H_10_O_4_ DMP	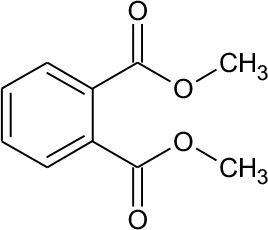	① ② ③
M1	211	C_10_H_10_O_5_ Dimethyl hydroxybenzoate	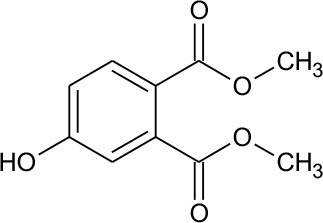	② ③
M2	152	C_8_H_8_O_3_ Methyl-o-hydroxybenzoate	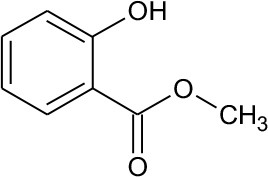	①
M3	179/156	C_8_H_12_O_3_ Methyl(2E,4E)-7-hydroxylhepta-2,4-dienoate	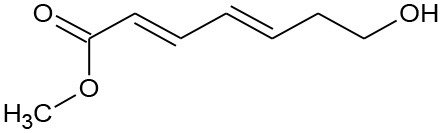	②
M4	155	C_8_H_10_O_3_ Methyl (2E,4E)-7-oxohepta-2,4-dienoate	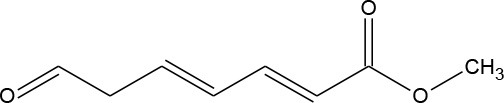	② ③

*①, UV system; ②, UV/TiO_2_ system; ③, UV-Vis/Bi_2_WO_6_ system*.

**Figure 4:**
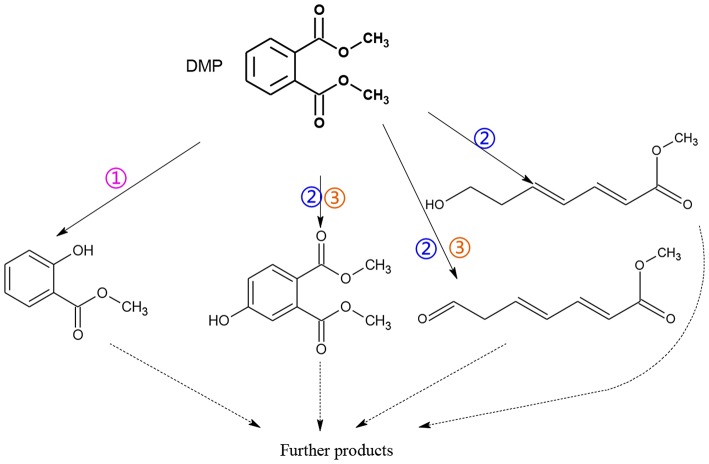
Proposed pathway for DMP degradation in different systems (① UV system; ② UV/TiO_2_ system; ③ UV-Vis/Bi_2_WO_6_ system).

As seen from Figure 3, only methyl-o-hydroxybenzoate was clearly detected by GC-MS in the photolysis systems after 300 min of irradiation, and the concentration continued to increase over time. However, more than one intermediates were analyzed in both photocatalytic systems: dimethyl hydroxybenzoate and one ring-opening byproduct methyl (2E,4E)-7-oxohepta-2,4-dienoate. Another ring-opening byproduct, methyl (2E,4E)-7-hydroxylhepta-2,4-dienoate, was also detected under the UV/TiO_2_ system. The degradation pathways of DMP in different systems were deduced by combining the byproducts and characteristics of each system (Figure 4): ① UV system: short wave ultraviolet directly cleaved the C–C bond of the aromatic ring and alphatic chain, resulting in the generation of a transitional product (DMP-), then the unstable DMP- reacted with OH- in the water and transformed into methyl-o-hydroxybenzoate; ② UV/TiO_2_ system: it has been reported that hydroxyl radicals were the main oxidative species in many studies of DMP photodegradation by TiO_2_ (Chen et al., 2009; Wang et al., 2018) and dimethyl hydroxybenzoate was the products of hydroxylation. Methyl (2E,4E)-7-oxohepta-2,4-dienoate and methyl (2E,4E)-7-oxohepta-2,4-dienoate were the product when hydroxyl attacked the carbon in the aromatic ring; ③ UV-Vis/Bi_2_WO_6_ system: similar with TiO_2_, methyl hydroxybenzoate was the hydroxylation product. However, the concentration of the byproduct methyl (2E,4E)-7-oxohepta-2,4-dienoate increased slowly from 0 to 300 min in UV/TiO_2_ system (Figure 3B), while the same compound increased rapidly over 30 min in the UV-Vis/Bi_2_WO_6_ system (Figure 3C), which might be due to hole oxidation playing an important role for Bi_2_WO_6_ (Wang et al., 2010, 2012).

#### DEP

**Figure 5:**
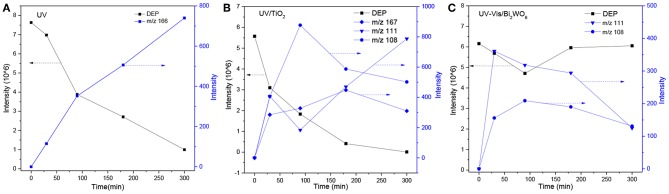
Time-dependent evolution profiles of DEP and its byproducts in different systems (**A**: UV; **B**: UV/TiO_2_; **C**: UV-Vis/Bi_2_WO_6_).

Under UV irradiation, DEP only produced one byproduct of ethyl salicylate (Figure 5A), similar to DMP. There are five byproducts under UV/TiO_2_ and two under UV-Vis/Bi_2_WO_6_ (Table 2). As shown in Figure 1E, the UV-Vis/Bi_2_WO_6_ system had almost no photoactivity to DEP, while UV/TiO_2_ has a strong activity to DEP, which could explain the differences in the kinds of byproduct and their intensities between Figures 5B,C. However, no ring-opening byproduct was detected by GC-MS in any degradation system for DEP, which might be due to its resistance to degradation, as discussed in section Removal of DMP, DEP, and DBP. Wang Z. et al. (2019) discussed the potential initial degradation pathways of DEP by computational analysis. The results indicated that the C–H bond of the alkyl chain directly connected to the ester bond had a lower net charge difference than other sites on alkyl side chains, especially the bond of the benzene ring. Combining the experimental results and the computational analysis, it is easy to understand why there were no ring-opening byproducts for DEP in any of the photocatalytic systems.

**Table 2:** GC-MS results for DEP and its byproducts.

**Byproduct**	**m/z**	**Formula**	**Possible structure**	**System**
DEP	222	C_12_H_14_O_4_ DEP	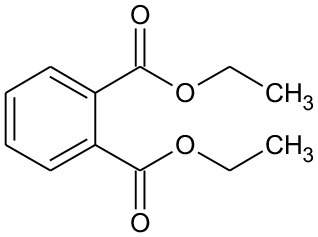	① ② ③
E1	166	C_9_H_10_O_3_ Ethyl salicylate	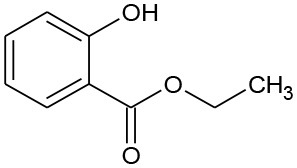	①
E2	194	C_9_H_10_O_4_ Mono-ethyl phthalate	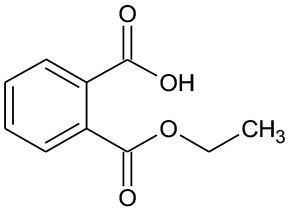	②
E3	167	C_8_H_6_O_4_ Phthalic acid	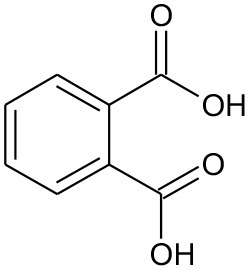	②
E4	149	C_8_H_4_O_3_, O-phthalic anhydride	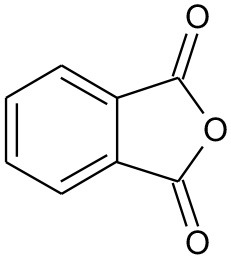	②
E5	111	C_6_H_6_O_2_ Benzenediol	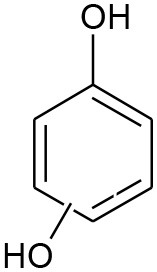	② ③
E6	108	C_6_H_4_O_2_ P-benzoquinone	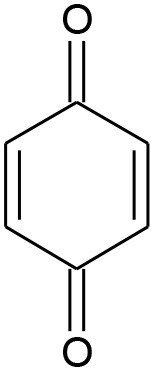	② ③

*① UV system; ② UV/TiO_2_ system; ③ UV-Vis/Bi_2_WO_6_ system*.

**Figure 6:**
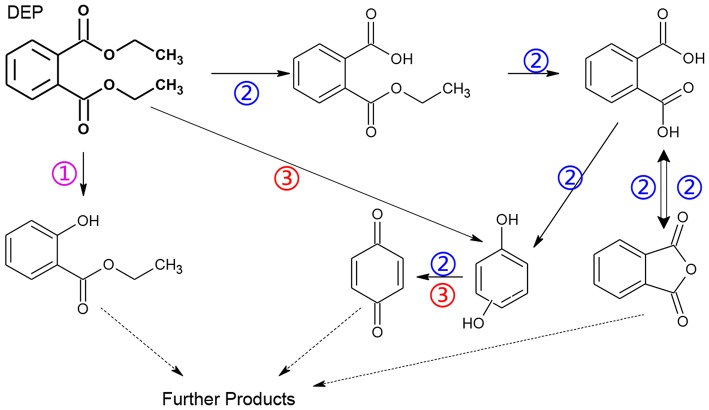
Proposed pathway for DEP degradation in different systems (① UV system; ② UV/TiO_2_ system; ③ UV-Vis/Bi_2_WO_6_ system).

The degradation pathways of DEP in all systems were proposed, as seen from Figure 6. Like the reaction mechanism of DMP in the photolysis system, ethyl salicylate was derived from the scission of the C–C bond of the aromatic ring and ethyl formate chain of DEP. As for the UV/TiO_2_ system, ethyl salicylate and phthalic acid were formed after sequential hydrolysis under the attack of hydroxyl radicals. Phthalic acid was then quickly transformed into o-phthalic anhydride, and further oxidation of phthalic acid produced benzenediol and p-benzoquinone (Zhang et al., 2016). UV-Vis/Bi_2_WO_6_ had almost no DEP-degradation ability, which might contribute to there being less or undetectable intermediates. There was no ring-opening.

#### DBP

The photolysis of DBP has different characteristics compared with DMP and DEP. Three byproducts were detected during the photolysis of DBP: butyl benzoate, benzoic acid, and butyl-o-hydroxybenzoate (Table 3). On the other hand, DBP was decomposed more thoroughly than DMP and DEP under the same conditions (Figure 1), as has been further proved by Wang Z. et al. (2019). However, similar to DMP, the ring-opening byproducts butyl (2E,4E)-7-oxohepta-2,4-dienoate was detected in both photocatalytic systems. Besides, identical with DEP, mono-butyl phthalate and p-benzoquinone were also detected in both photocatalytic systems. Butyl benzoate was the common byproduct of three degradation systems.

**Table 3:** GC-MS results of DBP and the byproducts.

**Byproduct**	**m/z**	**Formula**	**Possible structure**	**System**
DBP	278	C_16_H_22_O_4_ DBP	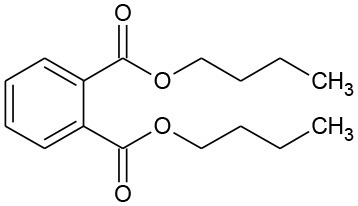	① ② ③
B1	194	C_11_H_14_O_3_ Butyl–o-hydroxybenzoate	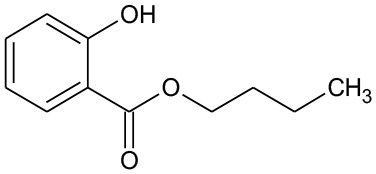	①
B2	219	C_11_H_16_O_3_ Butyl (2E,4E)-7-oxohepta-2,4-dienoate	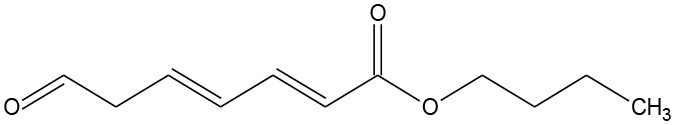	② ③
B3	221	C_11_H_14_O_4_ Mono-butyl phthalate	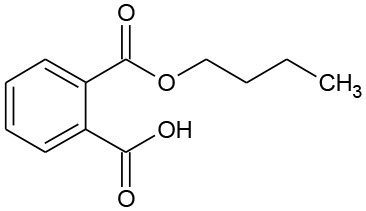	② ③
B4	177	C_11_H_14_O_2_ Butyl benzoate	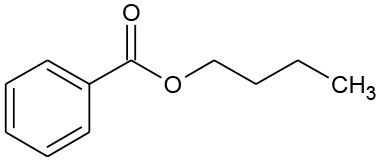	① ② ③
B5	122	C_7_H_6_O_2_ Benzoic acid	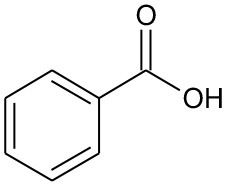	① ②
B6	111	C_6_H_4_O_2_ P-benzoquinone	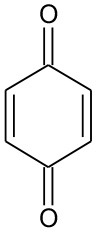	② ③

*① UV; ② UV/TiO_2_; ③ UV-Vis/Bi_2_WO_6_*.

**Figure 7:**
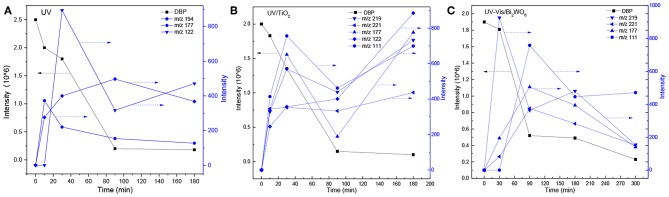
Time-dependent evolution profiles of DBP and its byproducts in different systems (**A**: UV; **B**: UV/TiO_2_; **C**: UV-Vis/Bi_2_WO_6_).

**Figure 8:**
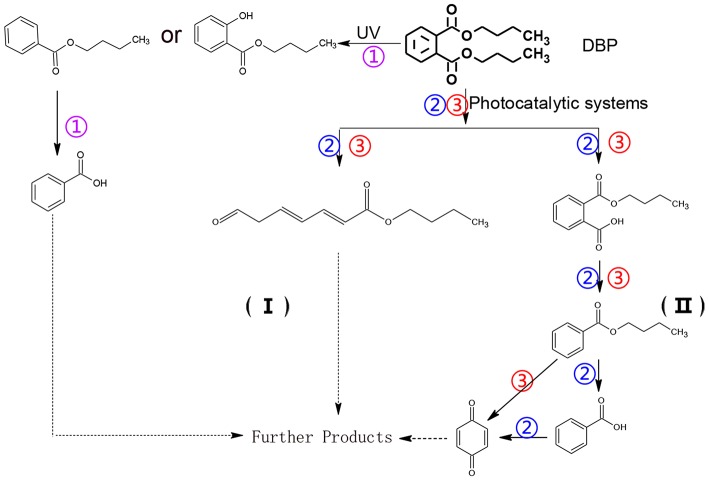
Proposed pathway for DBP degradation in different systems (① UV system; ② UV/TiO_2_ system; ③ UV-Vis/Bi_2_WO_6_ system).

The generation of butyl-o-hydroxybenzoate and butyl benzoate in the photolysis is according to Reaction (2) because of the attack of carbon from the aromatic ring by high-energy photons. Compared with butyl benzoate, butyl-o-hydroxybenzoate is the major product, as seen from Figure 7A. The further oxidation and hydrolysis of butyl benzoate produced benzoic acid. There were two pathways of DBP degradation in the UV/TiO_2_ system (Figures 7B, 8). First, similar to DMP, butyl (2E,4E)-7-oxohepta-2,4-dienoate was a ring-opening product when hydroxyl attacked the carbon of the aromatic ring. Second, the hydrolysis and decarboxylation generated mono-butyl phthalate and butyl benzoate, respectively, as has been proved by Zhang et al. (2019). Further products of benzoic acid and p-benzoquinone were derived from the oxidation and structural transformation. The rapid generation of the byproduct butyl (2E,4E)-7-oxohepta-2,4-dienoate during the first 30 min proved direct hole oxidation, as for DMP, in the UV-Vis/Bi_2_WO_6_ system (Figures 7C, 8). On the other hand, the reaction pathway of hydroxyl radicals with DBP was similar to the UV/TiO_2_ system. Mono-butyl phthalate, butyl benzoate, and p-benzoquinone were generated after sequential hydrolysis, decarboxylation, oxidation, and transformation.

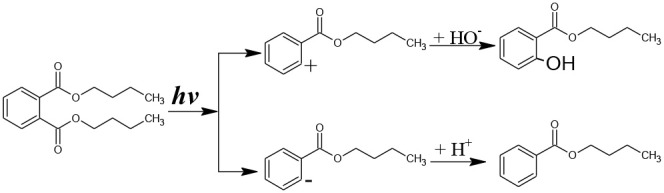



## Conclusions

The typical PAEs DMP, DEP, and DBP were photolyzed and photo-catalyzed under the conditions of UV, UV/TiO_2_, and UV-Vis/Bi_2_WO_6_. The ability of each system to degrade the chosen PAEs is in the following order: UV/TiO_2_ > UV > UV-Vis/Bi_2_WO_6_. The decomposition of PAEs obeys the rule of aliphatic chain length. The longer the aliphatic chain, the easier the removal of PAEs. The three typical PAEs exhibit almost the same degradation pathway under the UV system because of their similar byproduct, alkyl-o-hydroxybenzoate. Reactive species attacked mainly the aromatic ring of DMP in both photocatalytic systems, while the aliphatic chain was the major attack target in DEP. However, DBP may undergo both of the above two degradation modes.

## Data Availability Statement

The raw data supporting the conclusions of this manuscript will be made available by the authors, without undue reservation, to any qualified researcher.

## Author Contributions

CW: conceptualization and writing—original draft preparation. TZ and QZ: investigation. CG and XL: writing review and editing SZ: data curation.

### Conflict of Interest

The authors declare that the research was conducted in the absence of any commercial or financial relationships that could be construed as a potential conflict of interest.
